# Contrasting vertical patterns of lignin phenols in karst and non-karst forest soils

**DOI:** 10.3389/fpls.2026.1963199

**Published:** 2026-09-30

**Authors:** Jiaxin Yang, Jun Peng, Yuan Zhao, Peilei Hu, Yali Ding

**Affiliations:** 1 School of Resources and Environmental Engineering, Anhui University, Hefei, China; 2 School of Geographic Engineering and Spatial Information, Chuzhou University, Chuzhou, China; 3 Huanjiang Observation and Research Station for Karst Ecosystem, Guangxi Key Laboratory of Karst Ecological Processes and Services, Institute of Subtropical Agriculture, Chinese Academy of Sciences, Changsha, China; 4 Changsha Natural Resources Comprehensive Investigation Center, China Geological Survey, Changsha, China; 5 School of Soil and Water Conservation, Beijing Forestry University, Beijing, China

**Keywords:** karst ecosystems, lignin phenols, lithology, soil organic carbon, soil profile

## Abstract

Plant-derived carbon is a major source of soil organic carbon (SOC), but how its content, composition, and controls vary with bedrock lithology and soil depth remains unclear. We quantified SOC and lignin phenols in surface (0-10 cm) and subsurface (10-20 cm) soils from karst and non-karst forests across southwestern China and examined their relationships with climatic, plant, soil, and geochemical attributes. SOC content did not differ between lithologies at either depth. However, total lignin phenol content was higher in non-karst surface soils but similar between lithologies in subsurface soils. Cinnamyl-type phenols were higher in non-karst soils at both depths, whereas syringyl- and vanillyl-type phenols were higher in karst subsurface soils. Total lignin phenol content increased with depth only in karst soils, driven by subsurface syringyl and vanillyl enrichment. Lignin phenol variation was more strongly related to soil and geochemical properties than to plant attributes. In karst soils, lignin phenols were negatively associated with temperature and precipitation at the surface but positively associated with total nitrogen and exchangeable calcium and magnesium in the subsurface. In non-karst soils, lignin phenols were negatively associated with precipitation only at the surface but positively associated with total nitrogen and reactive aluminum (hydr)oxides at both depths. These findings show that similar SOC contents can mask lithology-dependent differences in the composition and vertical distribution of plant-derived carbon and highlight the value of combining molecular biomarkers with soil geochemistry.

## 1 Introduction

Soil organic carbon (SOC) is a major component of the terrestrial carbon pool and supports nutrient retention, aggregate formation, and microbial activity (Kopittke et al., 2022). Measurements of bulk SOC provide a direct basis for quantifying soil carbon stocks, but may not fully capture variation in carbon sources and transformation processes. Current conceptual frameworks suggest that SOC persistence reflects interactions among organic matter, soil physicochemical conditions, and biological processes, rather than molecular recalcitrance alone (Lehmann and Kleber, 2015; Schmidt et al., 2011). Lignin phenols provide molecular-level indicators of plant-derived carbon inputs and their subsequent transformation in soil (Thevenot et al., 2010). The three major lignin phenol groups, including vanillyl (V), syringyl (S), and cinnamyl (C) phenols, are commonly used to trace vascular-plant inputs and to distinguish broad differences among plant tissue sources (Chen et al., 2021; Ma et al., 2018). Integrating lignin phenol composition with bulk SOC measurements can therefore reveal variation in plant-derived carbon cycling that is not apparent from bulk SOC content alone.

Parent material can influence soil carbon dynamics through its effects on soil development, hydrology, pH, nutrient availability, and reactive mineral phases (Li et al., 2024; Slessarev et al., 2022). Roots and rhizodeposits are major contributors to SOC formation, whereas root channels, preferential flow, and dissolved organic matter transport can redistribute plant-derived carbon to deeper soil layers (Rumpel and Kögel-Knabner, 2011; Sokol et al., 2019). Karst and non-karst settings provide contrasting geochemical and physical templates for soil carbon cycling (Hu et al., 2024). Karst landscapes are soluble and weather relatively rapidly, producing landscapes characterized by shallow, discontinuous soils, relatively high pH, and abundant exchangeable calcium (Ca) and magnesium (Mg) (Hu et al., 2024; Liu et al., 2011). By contrast, adjacent clastic rocks generally form deeper, more continuous soils that are more acidic and more strongly influenced by iron (Fe)- and aluminum (Al)-bearing mineral phases. In karst systems, shallow surface soils do not necessarily confine plant carbon inputs to the upper soil layer. Tree roots can penetrate soil-filled fissures, dissolution features, and fractured bedrock to access water and nutrients stored below the surface (Jiang et al., 2020; Liu et al., 2025; McCormick et al., 2021). Such rooting patterns may spatially separate aboveground litter and shallow herbaceous-root inputs from deeper woody-root inputs at the soil-rock interface and within fissures. Lignin phenols provide a useful molecular approach for detecting this vertical separation. V phenols occur broadly in vascular plants, S phenols are characteristic of angiosperms, and C phenols are particularly abundant in non-woody tissues and grasses (Thevenot et al., 2010). Accordingly, depth-dependent shifts in the relative distribution of these phenol groups can indicate changes in plant-derived carbon sources, although microbial transformation and mineral-associated retention may also modify the resulting signatures.

Lithology may also influence the fate of plant-derived compounds after they enter soil by shaping the geochemical mechanisms that promote their retention. In karst soils, Ca may enhance aggregate formation (Rowley et al., 2018; Shabtai et al., 2023). In more acidic non-karst soils, short-range-order and crystalline Fe- and Al-bearing phases can retain organic compounds through sorption, ligand exchange, and co-precipitation (Lalonde et al., 2012; Liao et al., 2022; Zhu et al., 2024). Previous work has shown that lithology regulates microbial necromass accumulation and mineral-associated carbon stabilization (Hu et al., 2024). Molecular evidence from karst topsoils further suggests that bedrock type can alter the distribution of plant lipids and lignin phenols among soil aggregate fractions (Lin et al., 2025). However, it remains unclear whether lignin phenols show lithology-dependent vertical distributions across co-located karst and non-karst forests, and whether the factors associated with their distribution shift among climate, vegetation, soil properties, and specific mineral pools across soil depths.

Here, we addressed this knowledge gap along a broad climatic gradient in subtropical southwestern China, where forests developed on karst and non-karst substrates occur in close proximity (Wang et al., 2019). We sampled karst and non-karst forest soils at 0–10 and 10–20 cm depths and quantified SOC, C-, S-, and V-type phenols, and total lignin phenols. We then assessed the relative contributions of climate, plant attributes (diversity and biomass), soil physicochemical properties (pH and total nitrogen), and soil geochemical attributes (exchangeable Ca and Mg, as well as Fe and Al (oxyhydr) oxides) to variation in lignin phenols. We tested three hypotheses. First, lignin phenols show lithology-dependent differences in plant-source composition and vertical distribution. Second, lignin phenol variation would be more closely associated with soil-related variables than with stand-level plant attributes, with climatic associations being most evident in surface soils. Third, the associations between lignin phenols and environmental variables would differ between lithologies and soil depths, with exchangeable Ca and Mg being more closely associated with lignin phenols in carbonate-derived soils and reactive Fe- and Al-(hydr)oxide pools showing stronger associations in acidic clastic-derived soils. By combining molecular indicators of plant-derived carbon with lithology-specific soil physicochemical properties and geochemical attributes, our study provides a framework for understanding how contrasting bedrock types shape the distribution of plant-derived carbon in heterogeneous subtropical landscapes.

## 2 Materials and methods

### 2.1 Study area and sampling design

The study was conducted along a latitudinal transect in southwest China (21.84–29.11° N, 106.95–108.75° E; **Figure 1**), spanning Chongqing, Guizhou, and Guangxi provinces. The region is dominated by extensive karst landscapes and experiences a typical subtropical monsoon climate, characterized by a wet season from April to September and a dry season from October to March. According to long-term records obtained from the China National Meteorological Information Center (http://data.cma.cn/), mean annual precipitation (MAP) ranges from 1165 to 1878 mm and mean annual temperature (MAT) from 11.5 to 21.7 °C across the transect. The broad climatic variation across the transect provides an excellent natural gradient for evaluating the relative roles of climate, plant properties, and soil physicochemical properties in affecting lignin phenol accumulation within contrasting karst and non-karst soil profiles. The geological settings differ markedly between the two landscape types. Karst sites are predominantly underlain by limestone, dolomite, or mixtures of these carbonate rocks (dominated by CaCO_3_ and CaMg(CO_3_)_2_), whereas non-karst sites are mainly developed on clastic rocks (such as sandstone and shale, rich in silicates and Fe/Al oxides). Karst sites were dominated by limestone-adapted tree species (e.g., *Quercus* and *Lindera* spp.), whereas non-karst sites were dominated by acid-tolerant species (e.g., *Castanopsis* and *Carpinus* spp.) (Hu et al., 2024).

**Figure 1:**
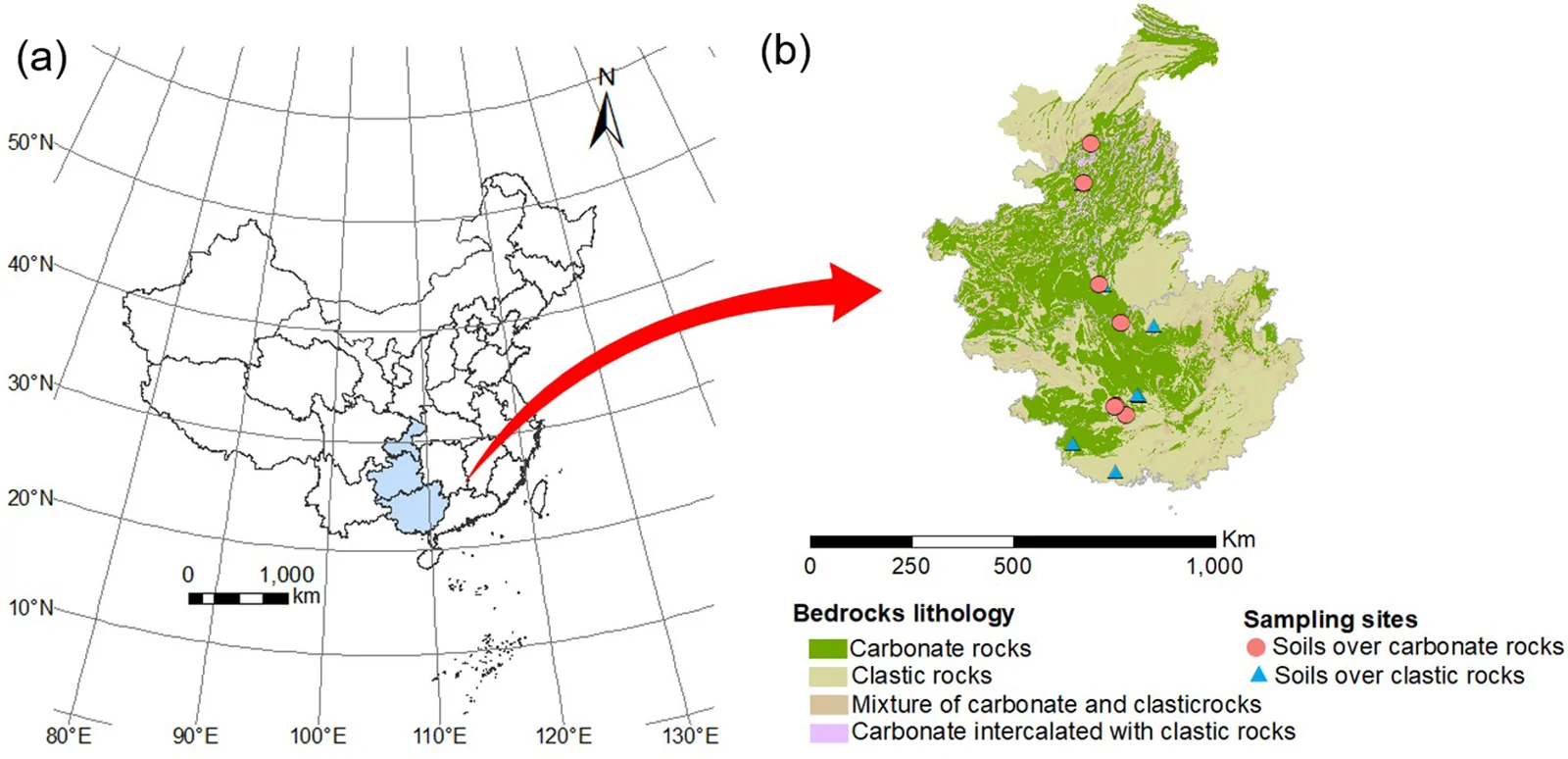
Spatial distribution of soil sampling sites in the karst region of southwest China. **(a)** The location of the study area within China; **(b)** Lithological composition and sampling points across the study area.

Field sampling was conducted in six representative locations, including Nanchuan (NC in Chongqing), Suiyang (SY in Guizhou), Dushan (DS in Guizhou), Huanjiang (HJ in Guangxi), Wuming (WM in Guangxi), and Longzhou (LZ in Guangxi). Within each region, five 20 × 30 m plots were established in karst forest and another five plots of the same size in non-karst forest, yielding 60 plots in total (6 regions × 2 lithologies × 5 plots). Plots were selected independently within each lithology. At the karst sites, soils were shallow and spatially discontinuous, with county-level mean soil depths ranging from 9 to 77 cm across the six regions, whereas soils at the paired non-karst sites were generally deeper than 50 cm. The five plots within each lithology were spatially distinct and were generally separated by more than 10 m but generally less than 1 km. To minimize the influence of vegetation types and topographic heterogeneity, all sampling sites were selected in naturally regenerated secondary forests with a stand age of approximately 60 ± 5 years following extensive deforestation during the late 1950s, with no major human disturbances since then. In addition, all plots were located on south or southeast-facing slopes with slope angles of 15–30° across an elevation gradient from 203–1523 m to reduce variation associated with microclimatic conditions. Following the removal of the surface litter layer, mineral soils were collected from two depth intervals (0–10 and 10–20 cm). Within each plot, twenty soil cores (38 mm in diameter) were randomly collected and homogenized to form one composite sample for each depth. Visible roots, plant residues, and gravel were manually removed before the soils were passed through a 2-mm sieve for subsequent physicochemical and biochemical analyses. The sieved soils were air-dried indoors before physicochemical and lignin phenol analyses.

Within each plot, all woody individuals were identified to species level, and the Shannon diversity index (H′) was calculated to characterize tree species diversity as $H^{\prime }=-\sum_{i=1}^{S}pi\ln pi$, where *pi* is the relative abundance of species *i* and S is the total number of species. Aboveground biomass (AGB) was estimated for each tree using species-appropriate allometric equations based on diameter at breast height (DBH), and plot-level AGB was obtained by summing the biomass of all trees within each plot and expressed on an area basis (kg m^-2^).

### 2.2 Soil physicochemical analyses

Soil pH was measured using a pH meter (FE20-FiveEasy™, Mettler-Toledo, Schwerzenbach, Switzerland) in a 1:2.5 soil-to-water suspension. SOC and total nitrogen (TN) were determined using a carbon–nitrogen elemental analyzer (vario MAX Cube, Elementar Analysensysteme GmbH, Hanau, Germany). Exchangeable Ca and Mg (Ca_ex_ and Mg_ex_) were extracted with 1 mol L^–1^ ammonium acetate at pH 7.0 and quantified by inductively coupled plasma–optical emission spectrometry (Agilent 5110 ICP-OES, Agilent, Santa Clara, USA). The same extraction procedure was applied to karst and non-karst soils to maintain a consistent operational definition and enable methodologically comparable measurements between lithologies. Because ammonium acetate may also mobilize Ca and Mg from carbonate-associated pools in karst soils, Ca_ex_ and Mg_ex_ are interpreted as operationally defined ammonium-acetate-extractable pools rather than as measurements of exclusively exchangeable cations.

Oxalate-extractable Al and Fe (Al_o_ and Fe_o_) and dithionite-extractable Al and Fe (Al_d_ and Fe_d_) were determined using standard operationally defined extraction procedures to characterize mineral pools differing broadly in crystallinity and reactivity. These extractions do not directly determine Fe oxidation state, redox dynamics, reactive oxygen species production, or Fenton-like reactivity; therefore, the resulting values were treated as operationally defined mineral-pool proxies rather than direct measures of oxidative or preservative mechanisms.

### 2.3 Lignin phenol analysis

Lignin phenols were regarded as reliable biomarkers of plant-derived carbon in soil (Kögel-Knabner, 2017; Thevenot et al., 2010). Their primary monomers include V-type, S-type, and C-type phenols. The sum of these three monomer types constitutes the total lignin phenol content.

The acid-to-aldehyde ratios of V- and S-type phenols, (Ad/Al)_v_ and (Ad/Al)_s_, were calculated as the ratios of vanillic acid to vanillin and syringic acid to syringaldehyde, respectively, and used as complementary indicators of lignin side-chain oxidation (Hedges et al., 1988; Thevenot et al., 2010). Lignin phenols were quantified using the alkaline copper oxide (CuO) oxidation method followed by solid-phase extraction and gas chromatography (Otto and Simpson, 2006; Pisani et al., 2015). Briefly, 0.5 g of air-dried soil was weighed into a PTFE digestion vessel, to which 0.5 g of CuO, 0.1 g of ferrous ammonium sulfate, and 0.05 g of glucose were added. Then, 10 mL of 2 mol L^–1^ NaOH solution and 50 μL of ethyl vanillin solution (1 mg mL^–1^ in 2 mol L^–1^ NaOH), used as the internal standard, were added. The mixture was subjected to alkaline oxidation at 182 °C for 2.5 h in a digestion bomb to depolymerize lignin into phenolic monomers. After cooling, the digest was transferred to a 50 mL centrifuge tube and centrifuged at 6000 rpm for 8 min. The pH of the supernatant was adjusted to 1.8–2.2 with 6 mol L^–1^ HCl. After standing in the dark for 1 h, the sample was centrifuged again at 6000 rpm for 8 min and filtered through a 0.45 μm nylon membrane. Before sample loading, the C_18_ solid-phase extraction cartridge was conditioned sequentially with 3 mL of ethyl acetate, 3 mL of methanol, and 5 mL of ultrapure water. After loading, the cartridge was vacuum-dried and stored in the dark in a desiccator for at least 1 week. The target compounds were eluted with 5.5 mL of ethyl acetate in two portions (4.0 and 1.5 mL). The eluate was evaporated to dryness under nitrogen at 30 °C. The dried residue was derivatized with 50 μL of pyridine and 100 μL of BSTFA at 60 °C for 10 min. Finally, the derivatized lignin phenols were analyzed using an Agilent 8890 gas chromatograph equipped with a flame ionization detector (GC-FID; Agilent Technologies, USA) and a DM-5 capillary column (30 m × 0.25 mm × 0.25 μm). Nitrogen was used as the carrier gas at a constant flow rate of 1.5 mL min^-^¹. The injector and detector temperatures were both set at 300 °C, with a 1 μL injection volume and a split ratio of 10:1. The oven temperature program was as follows: initial temperature of 100 °C, increased to 140 °C at 8 °C min^–1^, then to 170 °C at 4 °C min^-^¹ and held for 5 min, followed by an increase to 300 °C at 10 °C min^-^¹ and held for 4 min. Individual lignin phenol monomers were identified by matching their retention times with those of authentic standards. Three derivatized calibration levels (50, 100, and 150 μg mL^–1^), each containing ethyl vanillin as the internal standard, were analyzed. Relative response factors were calculated from the analyte-to-internal-standard peak-area and concentration ratios, and the concentrations of individual lignin phenol monomers were quantified using the internal-standard relative-response-factor method (Ma et al., 2018).

### 2.4 Statistical analysis

Before analysis, variable distributions were inspected, and model residuals were evaluated for normality and homogeneity of variance. Variables were natural-log-transformed when necessary to improve model assumptions. Linear mixed-effects models were used to test the effects of lithology, soil depth, and their interaction on SOC, V-, S-, and C-type phenol contents, total lignin phenol content, and soil physicochemical properties. Lithology (karst vs. non-karst), soil depth (0–10 cm vs. 10–20 cm) and their interaction were included as fixed effects, while site was included as a random intercept. Models were fitted using the “lmer” function in the “lme4” and “lmerTest” packages in R. Type III analysis of variance was used to evaluate the main effects of lithology, soil depth, and their interaction. Planned *post hoc* comparisons were conducted using estimated marginal means from the “emmeans” package. Specifically, we compared karst and non-karst soils within each soil depth, and compared the two soil depths within each lithology. Statistical significance was assessed at *p* < 0.05.

To quantify the relative contributions of different environmental factors to soil lignin phenols, we conducted hierarchical partitioning analysis based on adjusted R^2^. Predictor variables were grouped *a priori* into four categories: climate (MAP and MAT), plant attributes (Shannon diversity and AGB), soil physicochemical properties (pH and TN), and soil geochemical attributes (Ca_ex_, Mg_ex_, Al_o_, Fe_o_, Al_d_ and Fe_d_). Analyses were performed separately for each lithology and soil depth. For each subset, all possible combinations of the four predictor groups were fitted using linear models, with lignin phenols as the response variable. The independent contribution of each predictor group was calculated as its average incremental contribution to adjusted R^2^ across all model subsets. This approach partitions the total explained variation while accounting for shared explanatory power among predictor groups. The relative contribution of each group was calculated as its independent contribution divided by the sum of independent contributions across all predictor groups. Statistical significance was assessed using permutation tests, in which lignin phenols were randomly permuted 999 times and the hierarchical partitioning procedure was repeated. Permutation-based *p* values were calculated as the proportion of permuted independent contributions greater than or equal to the observed contribution. To evaluate the sensitivity of the estimated climate contribution, we conducted a complementary variance-partitioning analysis for each lithology depth subset. The pure climate adjusted R^2^ was quantified after conditioning on measured non-climate predictors and PCNM spatial eigenvectors. The analysis was repeated using MAT alone, MAP alone, MAT + MAP, and climate PC1 as alternative climate specifications.

Linear regressions were fitted separately for each lithology and soil depth to examine associations between lignin phenol content and individual climate and soil variables. Bivariate ordinary least-squares regression was selected because the response and predictor variables were continuous and the objective was to quantify the direction and strength of approximately linear associations. Its suitability was evaluated using scatterplots and residual diagnostics for linearity, residual normality, homogeneity of variance, and influential observations, and the resulting relationships were interpreted as descriptive rather than causal. For visualization, fitted regression lines and 95% confidence intervals were shown only for significant relationships (*p* < 0.05). To evaluate whether the wide numerical ranges and right-skewed distributions of Ca_ex_, Mg_ex_, Fe_o_, and Al_d_ affected the fitted relationships, these predictors were log10-transformed and the regressions were refitted. The original- and log-transformed models were compared using R^2^, RMSE, AIC, and residual diagnostics. Potential multicollinearity among environmental predictors was evaluated using pairwise Spearman correlations and variance inflation factors (VIFs), with |ρ| ≥ 0.70 and VIF > 5 indicating potentially important collinearity.

## 3 Results

### 3.1 SOC and lignin phenol contents in karst and non-karst soils

SOC content did not differ significantly between karst and non-karst soils at either soil depth (**Figure 2**). At 0–10 cm, mean SOC content was 57.25 g kg^–1^ in karst soils and 62.08 g kg^–1^ in non-karst soils; the corresponding values at 10–20 cm were 34.12 g kg^–1^ and 34.58 g kg^–1^, respectively (**Figure 2**). Within both lithologies, SOC content was significantly higher at 0–10 cm than at 10–20 cm (**Figure 2**).

**Figure 2:**
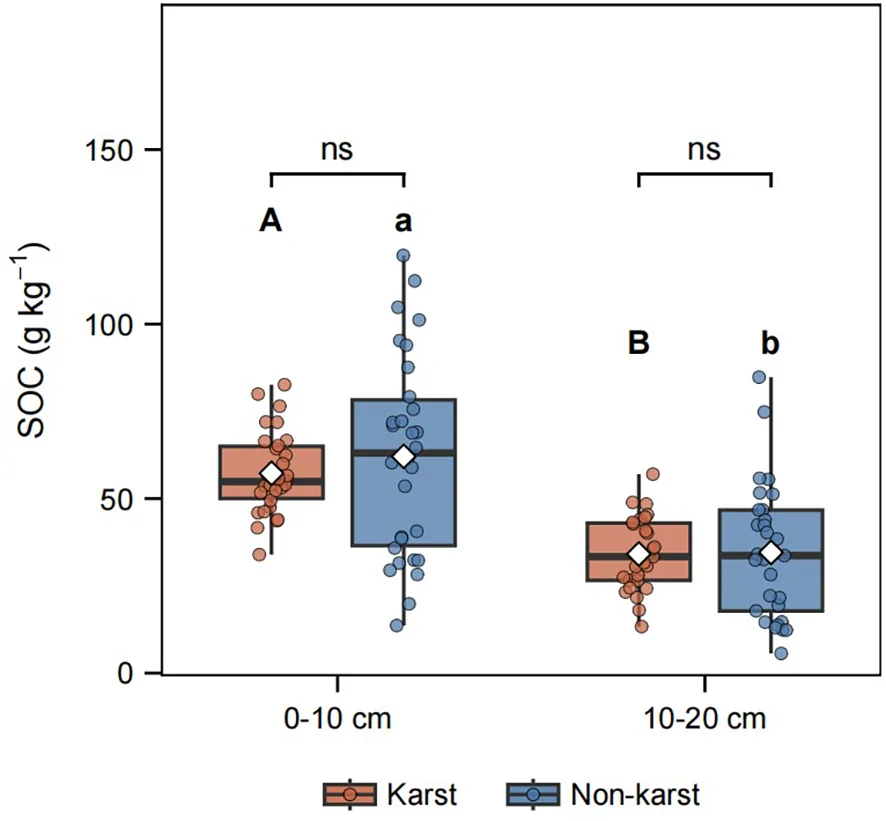
Soil organic carbon (SOC) between karst and non-karst soils at 0–10 and 10–20 cm depths. Points represent individual soil samples. Boxplots show medians, interquartile ranges and data distributions. Uppercase letters indicate significant depth differences within karst soils, whereas lowercase letters indicate significant depth differences within non-karst soils. ns indicates no significant difference (*p* > 0.05) between karst and non-karst soils within the same depth.

In contrast to bulk SOC, lignin phenols exhibited distinct lithology- and depth-dependent patterns (**Figure 3**). C-type phenol content was significantly higher in non-karst than in karst soils at both 0–10 cm (*p* < 0.001; **Figure 3A**) and 10–20 cm (*p* < 0.01; **Figure 3A**). S- and V-type phenol contents did not differ between lithologies at 0–10 cm but were significantly higher in karst soils at 10–20 cm (*p* < 0.05; **Figures 3B, C**). Total lignin phenol content was significantly higher in non-karst than in karst soils at 0–10 cm (*p* < 0.05) but did not differ between lithologies at 10–20 cm (**Figure 3D**).

**Figure 3:**
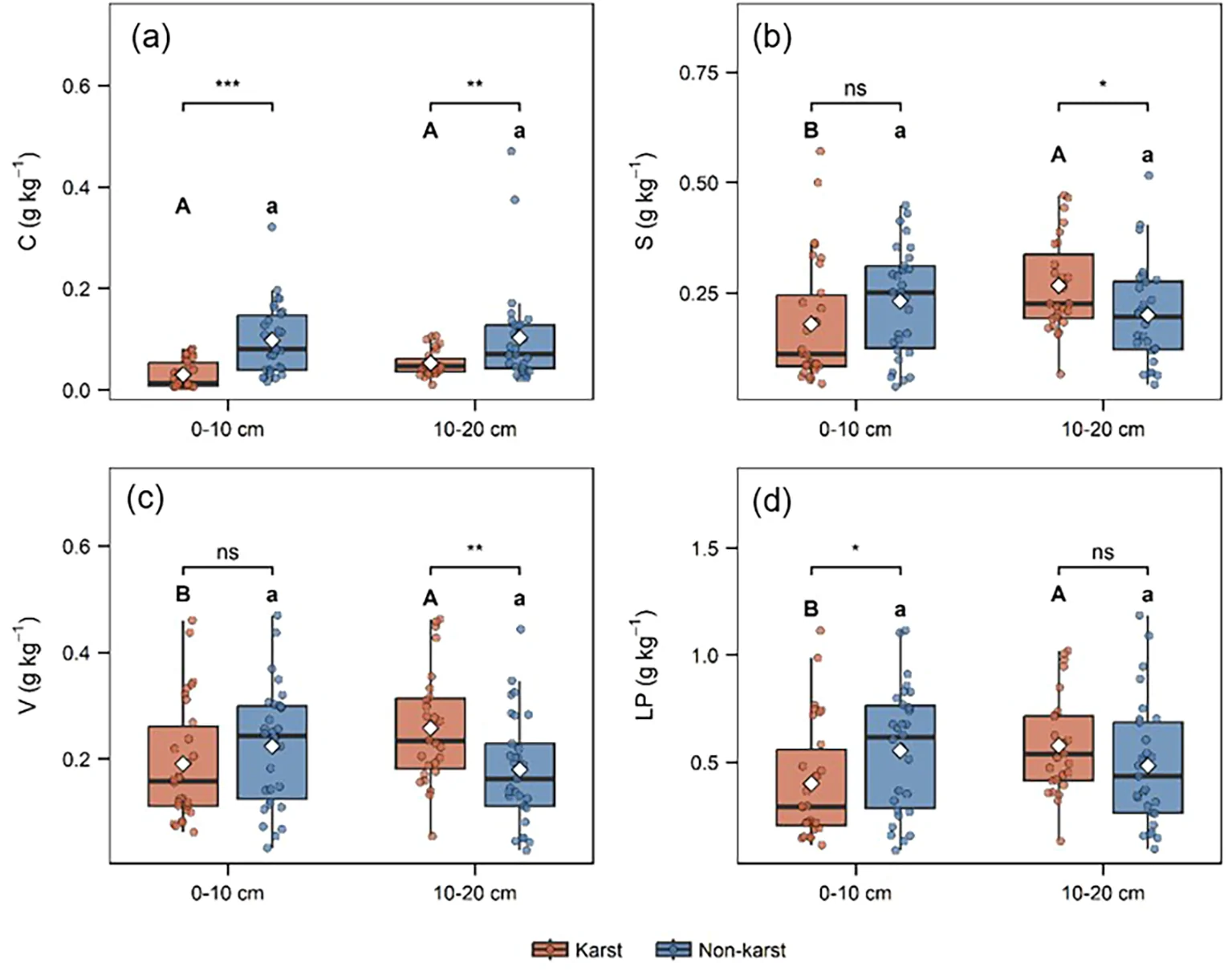
Lignin phenols between karst and non-karst soils. **(a)** C-type phenols, **(b)** S-type phenols, **(c)** V-type phenols, and **(d)** total lignin phenols (LP) in karst and non-karst forest soils at 0–10 and 10–20 cm depths. Points represent individual soil samples. Boxplots show medians, interquartile ranges and data distributions. Uppercase letters indicate significant depth differences within karst soils, whereas lowercase letters indicate significant depth differences within non-karst soils. Asterisks indicate significant differences between karst and non-karst soils within the same depth. *p < 0.05; **p < 0.01; ***p < 0.001; ns, not significant.

Within karst soils, S-type, V-type, and total lignin phenol contents were significantly higher at 10–20 cm than at 0–10 cm, whereas C-type phenol content did not vary significantly with depth (**Figure 3**). No significant depth-related differences in any lignin phenol measure were observed in non-karst soils (**Figures 3A–D**). Thus, the increase in total lignin phenol content with depth in karst soils was accompanied primarily by increases in S- and V-type phenols. Overall, lignin phenol composition and vertical distribution revealed lithology-dependent differences that were not apparent from bulk SOC content (**Figures 2**, **3**). To evaluate the relative abundance of lignin-derived phenols within the SOC pool, the ratio of total lignin phenol to SOC (LP/SOC) was calculated separately for each soil sample. Mean LP/SOC was numerically higher at 10–20 cm than at 0–10 cm in both lithologies, increasing from 0.73 ± 0.53% to 1.68 ± 0.40% in karst soils and from 0.89 ± 0.33% to 1.45 ± 0.47% in non-karst soils (**Supplementary Table S2**). Across both lithologies, mean (Ad/Al)_V_ was lower at 10–20 cm than at 0–10 cm, whereas mean (Ad/Al)_S_ was higher in the deeper layer (**Supplementary Table S2**).

### 3.2 Relative contributions of environmental factors to variation in lignin phenols

The relative contributions of climate, plant attributes, soil physicochemical properties, and geochemical attributes to total lignin phenol content differed between lithologies and soil depths (**Figure 4**). Together, these predictor groups explained 60% and 71% of the variation in karst surface and subsurface soils, respectively, and 58% and 88% of the variation in the corresponding non-karst soils (**Figure 4**). In karst surface soils, soil physicochemical properties were the largest contributors to lignin phenol variation (22.6%, *p* < 0.01), followed by climate (18.7%, *p* < 0.05; **Figure 4A**). At 10–20 cm, the contribution of soil properties increased to 36.6% (*p* < 0.01; **Figure 4B**). In non-karst surface soils, geochemical attributes made the largest independent contribution (31.8%, *p* < 0.05), followed by soil properties (20.7%, *p* < 0.05; **Figure 4C**). At 10–20 cm, soil physicochemical properties accounted for 65.7% of the variation (*p* < 0.01; **Figure 4D**). Overall, variation in lignin phenols was associated primarily with soil properties rather than plant attributes. In the original hierarchical partitioning, the climate-associated fraction was largest in karst 0–10cm soils, whereas soil geochemical attributes made their largest contribution in non-karst 0–10 cm soils. After spatial adjustment, the estimated pure climate contribution varied among alternative climate specifications, ranging from −4.89% to 7.39% (**Supplementary Figure S1**).

**Figure 4:**
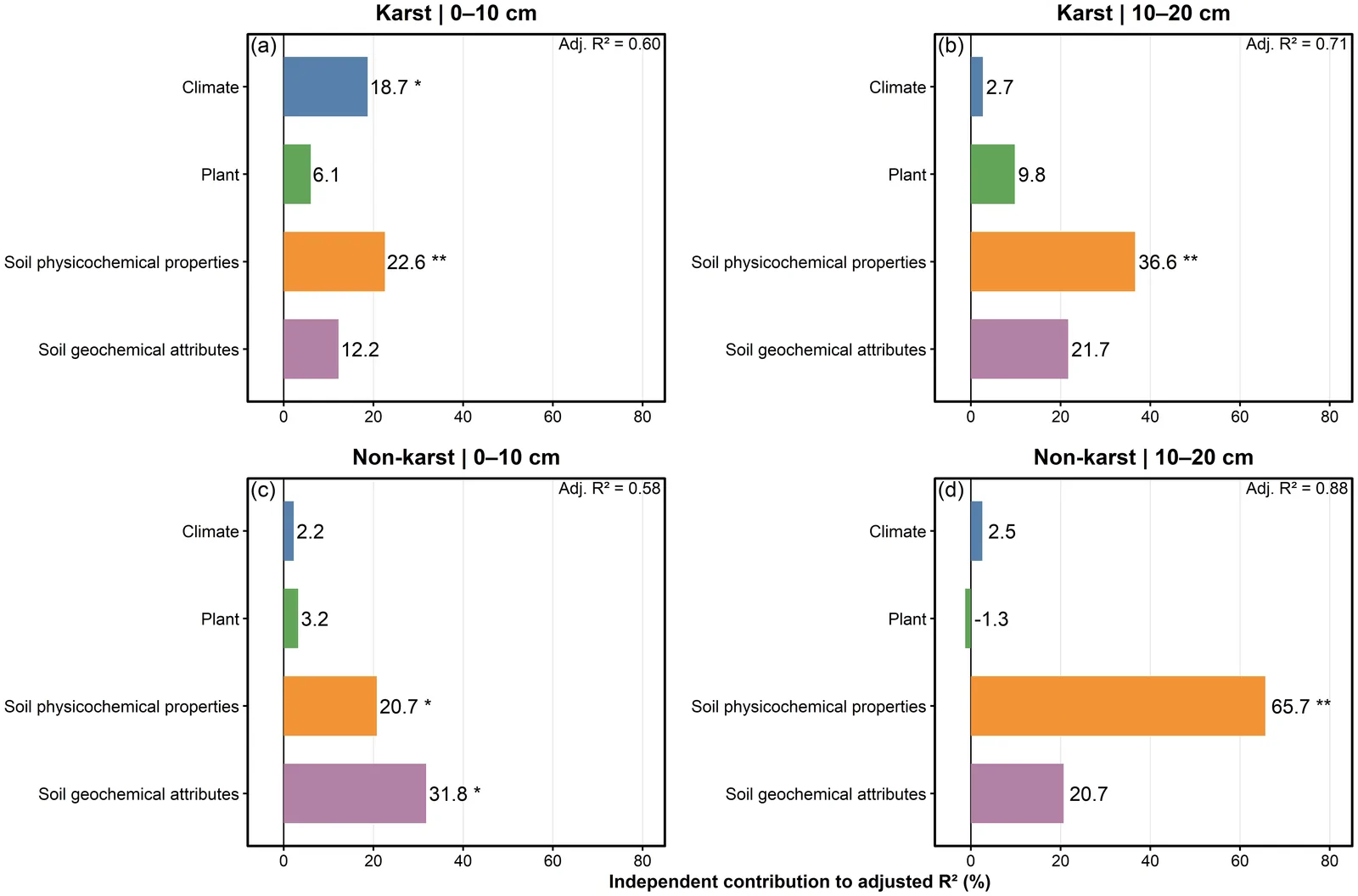
Relative contributions of climate, plant attributes, soil physicochemical properties, and soil geochemical attributes to soil lignin phenols in **(a)** karst soils at 0-10 cm, **(b)** karst soils at 10-20 cm, **(c)** non-karst soils at 0-10 cm, and **(d)** non-karst soils at 10-20 cm. Hierarchical partitioning was used to quantify the independent contributions of four predictor groups: climate (MAP and MAT), plant attributes (Shannon diversity and AGB), soil physicochemical properties (pH and TN), and soil geochemical attributes (Ca_ex_, Mg_ex_, Al_o_, Fe_o_, Al_d_, and Fe_d_). Bars represent the independent contribution of each predictor group to adjusted R2. *p < 0.05; **p < 0.01.

### 3.3 Associations of lignin phenols with climatic and soil factors

Plant attributes and soil geochemical attributes across lithologies and soil depths are summarized in **Table 1**. Plant Shannon diversity and AGB were both significantly lower in karst forests than in non-karst forests (*p< 0.01*; **Table 1**). For soil chemical properties, karst soils exhibited significantly higher Ca_ex_, Mg_ex_, TN and pH than non-karst soils across both 0–10 cm and 10–20 cm depths (*p< 0.01*; **Table 1**). In particular, Ca_ex_ and Mg_ex_ concentrations were substantially higher in karst soils at both depths. Regarding iron and aluminum oxides, Fe_d_ was significantly higher in karst soils at both depths, whereas Fe_o_ was lower in karst soils than in non-karst soils (*p< 0.05*; **Table 1**). Al_o_ was significantly lower in karst surface soils, whereas Al_d_ was significantly higher in karst surface soils (*p< 0.01*; **Table 1**). Across soil profiles, TN, Ca_ex_, and Al_d_ generally decreased with soil depth, whereas Fe_o_ and Al_o_ exhibited a pronounced increase from surface to subsurface soils in both lithologies (**Table 1**).

**Table 1:** Plant and soil properties between karst and non-karst soils.

Variable (unit)	Depth (cm)	Karst	Non-karst
Shannon diversity	–	2.62 ± 0.14**	3.06 ± 0.06
AGB (kg m^-2^)	–	10.28 ± 0.80***	15.47 ± 1.14
pH	0–10	6.56 ± 0.11^b^***	4.22 ± 0.06^b^
	10–20	6.74 ± 0.15^a^***	4.69 ± 0.13^a^
TN (g kg^-1^)	0–10	4.55 ± 0.13^a^**	3.68± 0.29^a^
	10–20	3.55 ± 0.17^b^***	2.62 ± 0.19^b^
Ca_ex_ (cmol kg^-1^)	0–10	34.03 ± 2.68^a^***	2.02 ± 0.44^a^
	10–20	19.45 ± 2.08^b***^	1.93 ± 1.11^a^
Mg_ex_ (cmol kg^-1^)	0–10	5.47 ± 0.68^a***^	0.61 ± 0.13^a^
	10–20	4.51 ± 0.59^b***^	0.53 ± 0.29^a^
Al_o_ (g kg^-1^)	0–10	4.58 ± 0.34^b^**	7.12 ± 0.89^b^
	10–20	9.33 ± 0.56^a^	10.47 ± 1.27^a^
Fe_o_ (g kg^-1^)	0–10	4.93 ± 0.63^b*^	8.23 ± 1.13^b^
	10–20	11.28 ± 1.09^a^***	18.21 ± 2.02^a^
Al_d_ (g kg^-1^)	0–10	18.90 ± 0.97^a^***	13.58 ± 1.80^a^
	10–20	7.94 ± 0.55^b^	5.92 ± 0.81^b^
Fe_d_ (g kg^-1^)	0–10	42.51 ± 3.40^a^***	23.83 ± 2.64^a^
	10–20	40.16 ± 3.78^a^***	23.27 ± 2.75^a^

Values are means ± standard errors. Plant Shannon diversity and aboveground biomass (AGB) were compared between karst and non-karst sites. Soil pH, total nitrogen (TN), exchangeable Ca and Mg (Ca_ex_ and Mg_ex_), oxalate-extractable Al and Fe (Al_o_ and Fe_o_), and dithionite-extractable Al and Fe (Al_d_ and Fe_d_) were compared between karst and non-karst soils within each soil depth and between soil depths within each lithology. Different lowercase letters indicate significant differences between depths within the same lithology (*p* < 0.05). Asterisks indicate significant differences between karst and non-karst sites within a given soil depth, or between the two forest-site groups for plant attributes: *p < 0.05; **p < 0.01; and ***p < 0.001.

The environmental variables associated with total lignin phenol content differed between lithologies and soil depths (**Figure 5**). In karst surface soils, total lignin phenol content was negatively associated with MAP, MAT, pH, Ca_ex_, Fe_d_, and Al_d_ (*p* < 0.05; **Figure 5A**). At 10–20 cm, total lignin phenol content was positively associated with TN, Ca_ex_, and Mg_ex_ (*p<* 0.05), but negatively associated with Fe_o_ (*p* < 0.05; **Figure 5A**). These results show that climatic associations were concentrated in karst surface soils, whereas associations with TN and exchangeable base cations were more evident in subsurface soils (**Figure 5A**).

**Figure 5:**
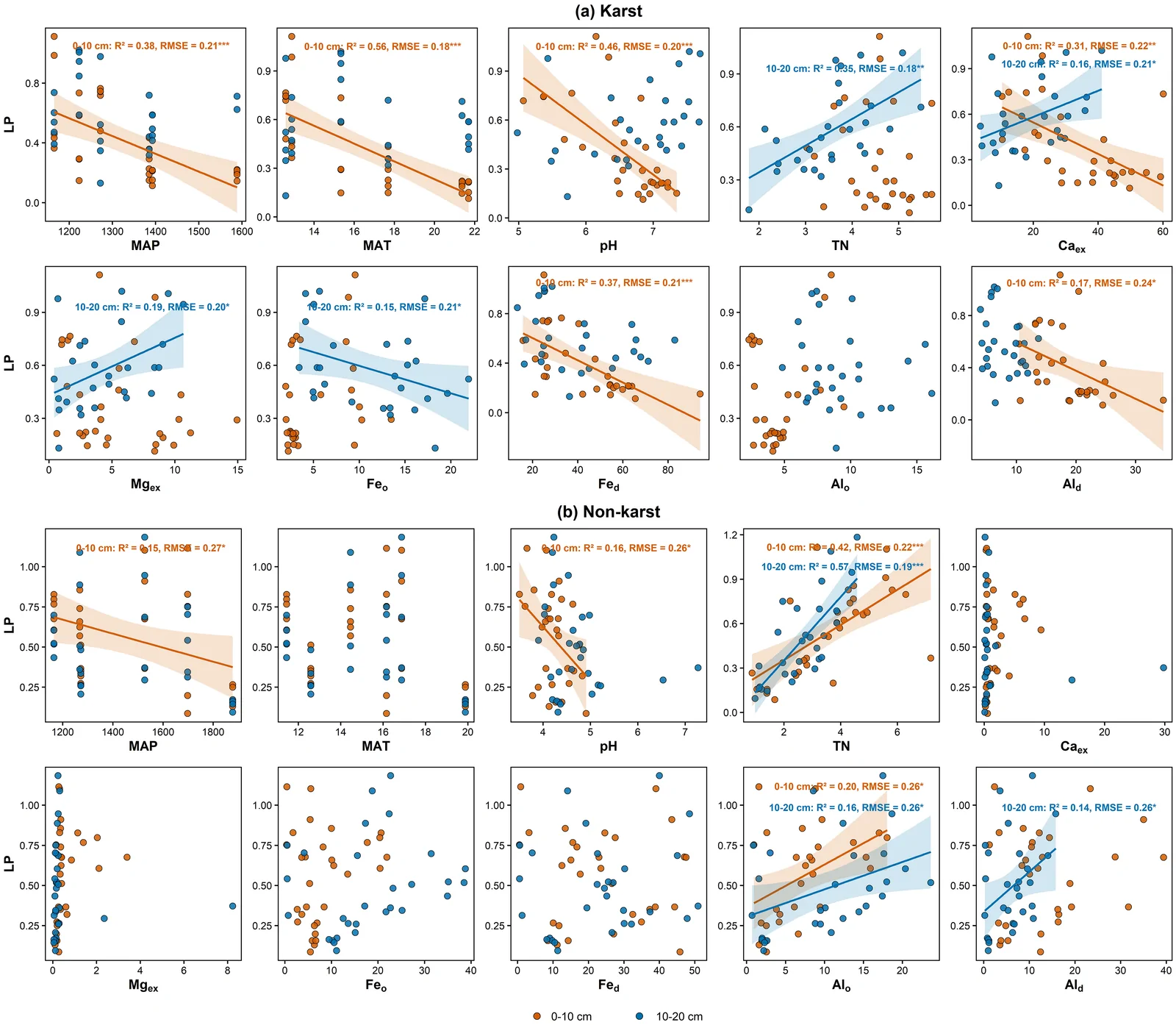
Relationships between soil lignin phenols and climatic and soil variables in **(a)** karst and **(b)** non-karst soils. Linear relationships between soil lignin phenols (LP) and MAP, MAT, pH, TN, Ca_ex_, Mg_ex_, Fe_o_, Fe_d_, Al_o_, and Al_d_ were analyzed separately for the 0-10 and 10-20 cm soil layers. Points represent individual soil samples, with colors denoting soil depth. Regression lines and shaded 95% confidence intervals are shown only for significant relationships. *p < 0.05; **p < 0.01; ***p < 0.001.

In non-karst surface soils, total lignin phenol content was negatively associated with MAP and pH (*p* < 0.05) and positively associated with TN and Al_o_ (*p* < 0.05; **Figure 5B**). At 10–20 cm, total lignin phenol content was positively associated with TN, Al_o_, and Al_d_ (*p* < 0.05), whereas no significant relationships with MAP or MAT were detected (**Figure 5**). Thus, lignin phenol variation in non-karst soils was more consistently associated with TN and Al-related variables than with climatic factors. Maximum VIFs ranged from 21.96 to 73.69 among the four lithology depth subsets (**Supplementary Table S1**), indicating that relationships involving correlated predictors should not be interpreted as independent effects.

## 4 Discussion

### 4.1 Lithology affects lignin phenol content and composition but not SOC content

The absence of significant differences in SOC content between karst and non-karst soils at either depth, despite clear differences in lignin phenol content and composition, indicates that bulk SOC did not fully reflect lithology-related variation in plant-derived carbon (**Figures 2**, **3**). Bulk SOC integrates organic compounds derived from plants and microorganisms that have undergone different degrees of transformation and mineral association over varying timescales (Cotrufo et al., 2013; Lehmann and Kleber, 2015). In contrast, lignin phenols are derived mainly from vascular plants and therefore reflect changes in plant-derived carbon more specifically, although their content is also jointly determined by plant input, decomposition, transport, and retention (Hedges and Mann, 1979; Thevenot et al., 2010). Consequently, comparable SOC contents can coexist with contrasting lignin phenol signatures because changes in the composition of plant-derived carbon may occur without producing a detectable change in the total SOC pool.

This distinction was particularly evident in the surface soil, where total lignin phenol content was higher in non-karst than in karst soils despite their similar SOC contents. At 10–20 cm, total lignin phenol content did not differ between lithologies, but its composition remained distinct: S- and V-type phenols were more abundant in karst soils, whereas C-type phenols were consistently more abundant in non-karst soils. Lithology therefore influenced the distribution and composition of lignin-derived plant carbon even when its effect was not evident from bulk SOC. A similar decoupling has been reported across grassland soils, where lignin phenols responded to climate and decomposition differently from bulk SOC (Ma et al., 2018). The higher total lignin phenol content in non-karst surface soils thus indicates a stronger lignin-derived plant-carbon signal. Overall, these findings show that bulk SOC alone may overlook lithology-related changes in soil organic matter composition, whereas combining SOC with lignin phenol biomarkers provides a more sensitive assessment of how parent material influences plant-derived carbon in soil.

### 4.2 Lignin phenol content increases with depth in karst but not in non-karst soils

Unlike SOC, which declined with depth in both lithologies, total lignin phenol content increased from 0–10 to 10–20 cm in karst soils, primarily because of increases in S- and V-type phenols (**Figure 3**). C-type phenols did not show a corresponding increase, and none of the phenol groups showed a comparable vertical change in non-karst soils.

This contrasting vertical pattern may partly reflect differences in plant community composition between the two ecosystems. Non-karst sites contained abundant acid-tolerant tree and shrub species, including *Carpinus viminea*, *Pieris japonica*, *Rhododendron rivulare*, and *Eurya* spp. These differences in vegetation composition may be consistent with the higher surface C-type phenol concentrations and C/V ratios observed in non-karst soils. In contrast, karst ecosystems are dominated by deeply-rooting, calciphilous woody trees such as *Cryptocarya chinensis*, *Itoa orientalis, Platycarya strobilacea and Lindera communis* (Hu et al., 2024). V-type phenols occur widely in vascular-plant lignin, S-type phenols are characteristic of angiosperms, and C-type phenols are relatively abundant in grasses and other non-woody tissues (Hedges and Mann, 1979). These source interpretations should also consider the operational nature of CuO oxidation. Alkaline CuO oxidation depolymerizes lignin-containing organic matter and releases diagnostic phenolic products; the measured compounds therefore represent CuO-recoverable lignin-derived phenols rather than necessarily free phenols originally present in soil (Hedges and Ertel, 1982; Thevenot et al., 2010). Mineral association can further influence their analytical recovery.

The shallow and discontinuous soil mantle of karst landscapes, together with extensively fractured carbonate bedrock, may facilitate the transfer of root-derived carbon to deeper soil layers. Woody roots can grow along soil-filled fissures and penetrate weathered limestone and dolomite to access subsurface water and nutrients, and field observations in karst and other rocky landscapes have shown that root distributions are closely associated with bedrock fractures (McCormick et al., 2021; Nie et al., 2014; Schwinning, 2010). Turnover of these roots may supply lignin-rich residues directly to subsurface soils and soil–rock interfaces (Sokol et al., 2019). This mechanism is consistent with the increase in S- and V-type phenols from 0–10 to 10–20 cm in karst soils. V-type phenols occur widely in vascular-plant lignin, whereas S-type phenols are characteristic of angiosperms and C-type phenols are relatively abundant in grasses and other non-woody tissues (Hedges and Mann, 1979). The predominance of deeply rooted woody species in the karst forest may therefore contribute to the greater abundance of S- and V-type phenols in the deeper soil layer.

Published tissue-level data provide further context for this interpretation. In a subtropical karst forest in southwest China, organic carbon (OC)-normalized lignin phenol contents averaged 34.70 ± 16.50 mg g^–1^ OC in leaves and 26.70 ± 11.60 mg g^–1^ OC in litter (Qian et al., 2023). At Dinghushan, lignin phenol yields in fine roots of *Ardisia quinquegona* ranged from 16.30 to 41.80 mg g^–1^ C and were approximately threefold higher than those in leaves (Wang et al., 2015). Russell et al. (2021) similarly reported total V-, S-, and C-type phenol contents of 19.10–25.40 mg g^–1^ OC in fine roots, compared with 9.60–19.62 mg g^–1^ OC in leaves. Our soil means ranged from 7.26 to 16.82 mg g^–1^ OC, overlapping partly with reported leaf values but remaining generally below published fine-root concentrations. Although direct numerical comparisons among studies are affected by differences in vegetation and analytical procedures, these observations support the potential importance of belowground plant inputs to the lignin phenol pool in karst soils.

The higher LP/SOC values at 10–20 cm in both lithologies further suggest a greater relative contribution of lignin-derived plant carbon to SOC in the deeper layer (**Supplementary Table S2**). Roots of trees and shrubs are strongly concentrated within the upper soil profile, and the 10–20 cm layer therefore remains an important zone of belowground carbon input. At the same time, bulk SOC and microbial activity generally decrease with soil depth (Jobbágy and Jackson, 2000; Naylor et al., 2022). Continued root-derived inputs, together with a stronger decline in other SOC components, could consequently increase the relative abundance of lignin-derived phenols at 10–20 cm. Post-depositional processes may further modify this pattern. Downward transport, differential degradation of lignin components, and interactions with Ca- and Fe/Al-bearing mineral phases can influence the retention and composition of lignin-derived compounds after their incorporation into soil (Huang et al., 2019; Rowley et al., 2021; Rumpel and Kögel-Knabner, 2011; Thevenot et al., 2010). The observed depth variation in acid-to-aldehyde ratios is also consistent with differences in lignin transformation between soil layers (**Supplementary Table S2**). Because CuO oxidation releases diagnostic phenolic products from lignin-containing organic matter, the measured compounds are most appropriately interpreted as indicators of lignin-derived inputs and subsequent transformation rather than as intact lignin molecules. Overall, the subsurface enrichment of S- and V-type phenols in karst soils is consistent with an important contribution from woody belowground inputs, further modified by transport, degradation, and mineral-associated retention.

### 4.3 Lithology-specific soil environments influence lignin phenol distributions

The variation in lignin phenol content was more strongly associated with soil physicochemical and geochemical properties than with aboveground biomass or tree diversity (**Figure 4**). This pattern does not diminish the role of vegetation as the ultimate source of lignin. Rather, it indicates that stand-level vegetation properties alone are insufficient to explain how plant-derived lignin is distributed after entering soil. Aboveground biomass and tree diversity mainly describe current vegetation structure, whereas soil lignin phenols integrate plant inputs over time together with subsequent decomposition, vertical transport, and interactions with the soil matrix (Kleber et al., 2015; Rumpel and Kögel-Knabner, 2011). A similar decoupling between standing plant biomass and soil lignin phenols has been reported in grassland ecosystems (Ma et al., 2018). Our results therefore suggest a division of controls in which vegetation determines the potential supply of lignin-derived carbon, whereas the soil environment exerts a stronger influence on its subsequent distribution and persistence.

The dominant associations differed between karst soil layers. In surface soils, lignin phenol content was negatively associated with MAT and MAP (**Figure 5A**), consistent with climatic regulation of organic-matter decomposition and lignin-transforming microbial activity (Donnelly et al., 1990; Thevenot et al., 2010). Temperature and moisture influence fungal activity and oxidative enzymes involved in lignin degradation, although their effects depend strongly on local soil conditions (A'Bear et al., 2014; Asther et al., 1988; Baldrian et al., 2010; McClaugherty and Linkins, 1990). In contrast, lignin phenol content at 10–20 cm was positively associated with TN, Ca_ex_, and Mg_ex_. This shift from climatic associations near the surface to nutrient- and base-cation-related associations at depth suggests that the controls on lignin-derived carbon change along the soil profile. In karst soils, high Ca and Mg availability can promote aggregation and organo-mineral interactions, potentially favouring the retention of plant-derived compounds entering deeper horizons. Together with the deeper woody-root inputs discussed above, these edaphic conditions provide a plausible explanation for the accumulation of S- and V-type phenols in karst subsurface soils.

Non-karst soils showed a different geochemical signature. Soil properties explained 65.7% of the variation in lignin phenol content at 10–20 cm, whereas geochemical attributes accounted for a further 20.7% (**Figure 4D**). Lignin phenol content was positively associated with Al_o_ in surface soils and with Al_o_ and Al_d_ at 10–20 cm (**Figure 5B**), while no significant relationship with MAT or MAP was detected in the deeper layer. These relationships point to a greater role of Al-bearing mineral phases in regulating lignin-derived carbon in non-karst soils. Reactive Al and Fe phases can retain organic compounds through adsorption, surface complexation, and coprecipitation, thereby influencing both their abundance and persistence in soil (Heckman et al., 2013; Huang et al., 2019; Kögel-Knabner et al., 2008). Fe-mediated oxidative reactions may simultaneously contribute to lignin transformation (Liao et al., 2022; Naughton et al., 2023), providing a mechanistic basis for the observed variation in acid-to-aldehyde ratios (Hedges et al., 1988; Thevenot et al., 2010). The contrasting Ca-Mg associations in karst soils and Al-related associations in non-karst soils therefore indicate that different mineral environments may regulate lignin-derived carbon across the two lithological settings.

These lithology-specific relationships help explain why differences in plant-derived carbon were more evident in lignin phenol composition and vertical distribution than in bulk SOC. Recent studies likewise show that parent material can alter SOC composition through differences in mineralogy, aggregation, and cation availability. Lin et al. (2025) for example, linked aggregate structure, clay mineralogy, and Ca availability to SOC storage and plant-derived biomarkers across sandstone-, limestone-, and dolomite-derived karst soils. Other work in carbonate-derived soils has emphasized strong organo-mineral control over microbial-derived carbon (Hu et al., 2024), while Zhu et al. (2026) identified microbial necromass rather than lignin as the dominant predictor of recalcitrant SOC during karst vegetation restoration. These studies point to a common conclusion: lithology influences not simply how much carbon is stored, but which carbon components accumulate and through which soil processes. In our study, Ca- and Mg-related associations were strongest in karst subsurface soils, whereas Al-related associations were more pronounced in non-karst soils, indicating distinct geochemical pathways regulating the vertical distribution of plant-derived carbon.

### 4.4 Limitations

Several aspects of the proposed mechanisms warrant further testing. Root abundance and vertical root distribution were not quantified in this study. The observed lignin phenol pattern should therefore be considered consistent with, rather than direct evidence for, a greater contribution of woody-derived belowground inputs at this depth. Downward transport of dissolved or particulate plant-derived compounds, depth-dependent decomposition, and cation-mediated retention may also contribute to the observed enrichment. Root-resolved sampling across the soil-bedrock continuum will be needed to distinguish their relative contributions. In addition, our analyses identify consistent lithology- and depth-dependent associations between lignin phenols and soil environmental variables, but the relative contributions of plant inputs, microbial transformation, and mineral retention remain to be resolved experimentally. In particular, direct measurements of root and litter inputs, soil microbial processes, and mineral-associated lignin fractions would help determine how these pathways contribute to the observed depth patterns. Likewise, mineralogical and spectroscopic approaches could further clarify the roles of Ca-, Mg-, Fe-, and Al-bearing phases in lignin retention and transformation. Despite these uncertainties, the consistent contrasts between karst and non-karst soils across regions and soil depths show that lithological context is important for understanding the distribution of plant-derived carbon. Future studies integrating plant inputs, microbial processes, and mineral interactions across the soil-bedrock continuum will provide a more complete mechanistic picture of how lithology regulates the fate of lignin-derived carbon.

## 5 Conclusions

Although SOC content did not differ between karst and non-karst soils at either depth, lignin phenol content and composition exhibited clear lithology- and depth-dependent patterns. Non-karst soils contained more total lignin phenols in the surface layer, whereas total lignin phenol content increased with depth only in karst soils, primarily because of increases in S- and V-type phenols. These patterns indicate that lignin phenols revealed clearer lithological contrasts than bulk SOC and are consistent with a greater contribution of lignified woody tissues to deeper karst soils. Lignin phenol variation was more closely associated with soil physicochemical properties and geochemical attributes than with aboveground plant attributes. Climate-related covariation was observed mainly in karst surface soils, whereas Ca- and Mg-related associations were most evident in karst subsurface soils and Al (hydr)oxide-related associations were more evident in non-karst soils. Overall, these findings indicate that the composition and vertical distribution of plant-derived biomarkers differed between lithologies and that soil geochemical context is important for understanding the distribution and dynamics of plant-derived carbon.

## Data Availability

The original contributions presented in the study are included in the article/**Supplementary Material**. Further inquiries can be directed to the corresponding authors.
