# Supplementary material for: Contrasting vertical patterns of lignin phenols in karst and non-karst forest soils
Source: Front Plant Sci. 2026 Sep 30;17:1963199. doi: 10.3389/fpls.2026.1963199 (PMC13662109; doi:10.3389/fpls.2026.1963199)
Supplement: Supplementary file 1 [file Table1.docx]

***Supplementary Material***

**Table S1. Variance inflation factors (VIFs) of environmental predictors within each lithology soil depth subset.**

| **Variable** | **Karst 0–10 cm** | **Karst 10–20 cm** | **Non-karst 0–10 cm** | **Non-karst 10–20 cm** |
| --- | --- | --- | --- | --- |
| MAP | **7.39** | **18.58** | **19.03** | **27.08** |
| MAT | **21.96** | **27.81** | **19.67** | **24.25** |
| pH | **16.47** | **19.22** | 4.05 | **11.35** |
| Ca_ex_ | **10.26** | **11.12** | **17.81** | **8.45** |
| Mg_ex_ | 2.83 | **28.65** | **8.65** | **16.06** |
| TN | 2.54 | 2.66 | **8.25** | **11.17** |
| Fe_o_ | **6.56** | **19.78** | **5.80** | **11.72** |
| Fe_d_ | **14.62** | **20.80** | **14.62** | **29.92** |
| Al_o_ | 2.21 | **19.27** | **13.16** | **13.43** |
| Al_d_ | **8.02** | **22.67** | **22.94** | **73.69** |

VIFs were calculated using the full set of environmental predictors within each lithology–soil depth subset. Bold values indicate VIF > 5 and potentially important multicollinearity.

**Table S2. Soil organic carbon-normalized total lignin phenol yields (LP/SOC) and acid-to-aldehyde ratios within each lithology–soil depth subset.**

| Lithology | Depth (cm) | LP/SOC (%) | (Ad/Al)_V_ | (Ad/Al)_S_ |
| --- | --- | --- | --- | --- |
| Karst | 0–10 | 0.73 ± 0.53 | 0.37 ± 0.11 | 0.27 ± 0.13 |
| Karst | 10–20 | 1.68 ± 0.40 | 0.28 ± 0.06 | 0.47 ± 0.08 |
| Non-karst | 0–10 | 0.89 ± 0.33 | 0.25 ± 0.07 | 0.43 ± 0.11 |
| Non-karst | 10–20 | 1.45 ± 0.47 | 0.20 ± 0.05 | 0.54 ± 0.12 |

Values are means ± standard deviation (SD). LP/SOC is the SOC-normalized total lignin phenol (LP) yield, calculated separately for each sample and expressed as mg total lignin phenols per 100 mg SOC (numerically equivalent to %). (Ad/Al) V and (Ad/Al) S are the acid-to-aldehyde ratios of vanillyl- and syringyl-type phenols, calculated as vanillic acid/vanillin and syringic acid/syringaldehyde, respectively. Missing observations were excluded separately for each variable before group statistics were calculated; therefore, sample sizes may differ among variables and subsets.

**Figure S1. Sensitivity analysis of climate variable groupings.** Pure climate adjusted R² (%) explaining variation in soil lignin phenol content after spatial adjustment across different regions and soil depths: (a) Karst, 0–10 cm; (b) Karst, 10–20 cm; (c) Non-karst, 0–10 cm; and (d) Non-karst, 10–20 cm. Climate variable groupings on the y-axis include Mean Annual Temperature only (MAT only), Mean Annual Precipitation only (MAP only), the combination of both (MAT + MAP), and the first principal component of climate variables (Climate PC1).

**Fig. S2 Relationships between soil lignin phenols with log_10_-transformed soil variables in karst and non-karst soils.** Linear relationships between soil lignin phenols (LP) and log_10_-transformed soil variables (Ca_ex_, Mg_ex_, Fe_o_, and Al_d_) were analysed separately for (a) karst and (b) non-karst soils. Within each lithology, regressions were fitted separately for the 0–10 and 10–20 cm soil layers. Points represent individual soil samples, with colors denoting soil depth. Regression lines and shaded 95% confidence intervals are shown only for significant relationships. *, *p* < 0.05; ***, *p* < 0.001.
